# Optical Methods for Label-Free Detection of Bacteria

**DOI:** 10.3390/bios12121171

**Published:** 2022-12-15

**Authors:** Pengcheng Wang, Hao Sun, Wei Yang, Yimin Fang

**Affiliations:** 1 Jiangsu Province Key Laboratory of Anesthesiology, Xuzhou Medical University, Xuzhou 221004, China; 2 Key Laboratory of Cardiovascular & Cerebrovascular Medicine, School of Pharmacy, Nanjing Medical University, Nanjing 211166, China

**Keywords:** bacteria detection, dark-field microscopy, Raman spectroscopy, surface plasmon resonance, label-free, rapid detection

## Abstract

Pathogenic bacteria are the leading causes of food-borne and water-borne infections, and one of the most serious public threats. Traditional bacterial detection techniques, including plate culture, polymerase chain reaction, and enzyme-linked immunosorbent assay are time-consuming, while hindering precise therapy initiation. Thus, rapid detection of bacteria is of vital clinical importance in reducing the misuse of antibiotics. Among the most recently developed methods, the label-free optical approach is one of the most promising methods that is able to address this challenge due to its rapidity, simplicity, and relatively low-cost. This paper reviews optical methods such as surface-enhanced Raman scattering spectroscopy, surface plasmon resonance, and dark-field microscopic imaging techniques for the rapid detection of pathogenic bacteria in a label-free manner. The advantages and disadvantages of these label-free technologies for bacterial detection are summarized in order to promote their application for rapid bacterial detection in source-limited environments and for drug resistance assessments.

## 1. Introduction

Bacteria are the most abundant, widely distributed, diverse microorganisms in nature and of a special type. After a long period of natural evolution, bacteria have established complex antagonistic or symbiotic relationships with various species [1]. Although most of the bacteria are harmless, bacterial and viral infections account for approximately 70% of all human pathogenic diseases [2]. Bacterial pathogens can be obtained from food, water, animals, and even clinical settings including hospitals and other healthcare facilities. Pathogenic bacteria such as Salmonella, *Escherichia coli* (*E. coli*), Staphylococcus, etc. are the main causes of foodborne illness, which poses a constant threat to food safety. Bacterial infection is considered to be a common and costly global public health problem [3,4]. Bacteria not only cause some specific diseases in the host, but also act as opportunistic pathogens. When the host’s immunity is low, the immune barrier is destroyed, flora imbalance or bacterial translocation occurs, which releases many virulent factors causing the host infection [5,6]. Treatment with antibiotics is the most effective and frequently used solution to this problem. Nevertheless, with the increasing use of antibiotics, the emergence of bacterial resistance to antibiotics is rising, which reduces the effectiveness of antibiotics for bacterial infection treatment, leading to increasing morbidity, mortality, and medical costs. According to the World Health Organization, antibiotic resistance kills 700,000 people every year, and if this problem is not addressed, the number of deaths resulting from antibiotic resistance will increase to 10 million by 2050 [7]. At present, bacterial resistance has become an increasingly serious global challenge, as well as a worldwide concern to governments and society [8]. According to the U.S. Centers for Disease Control and Prevention, about 2.8 million infections in the U.S. each year are related to antimicrobial resistance, implying significantly increasing treatment times and costs as well as mortality from bacterial infections [9].

The effectiveness of antibiotic treatment can be largely retained with the rational use of antibiotics. Rapid identification of pathogens is particularly important in clinical diagnosis, not only to minimize risks to patients, but also to provide a basis for physicians to prescribe pathogen-specific antibiotics rather than broad-spectrum antibiotics to reduce irrational use of antibiotics. However, rapid bacterial detection is quite a challenging task due to the large variety of bacteria and severe interference from the complex matrix in the growth environment [10]. Traditional methods, such as bacterial culture, PCR, and enzyme-linked immunosorbent assay (ELISA) are frequently used, but these methods have their own disadvantages. The bacterial culture method is the golden standard method for bacterial detection, but it is quite time-consuming, and easily contaminated by non-target bacteria. Detection of some clinically relevant pathogens by this method can take up to five days to develop an adequate culture [11]. PCR is a molecular biology technique used to amplify specific nucleic acid fragments. It replicates nucleic acid exponentially at a very low concentration [12] to a detectable amount within hours. Therefore, it has been widely used in bacteria detection. However, contamination of the test sample and erroneous DNA amplification can lead to false positive or negative results. PCR is relatively expensive and takes hours which is not rapid enough for regular use in antibiotic prescription. Immunoassays rely on the specific reaction of antigens and antibodies and are also used for the detection of bacteria [13,14] but are less sensitive and require a large amount of clinical samples.

To overcome these difficulties, more sensitive and rapid methods for bacterial detection have been extensively studied. In recent years, applications based on biosensors, which are analytical devices that convert biological responses into measurable signals, have become increasingly widespread [15]. Such an application usually consists of three parts: (1) ligands attached to the surface of the biosensor to recognize the target through specific interactions; (2) a sensor that converts biometric identification generated on the sensor surface into quantifiable physical signals such as light, electricity, heat, and voltage, etc.; (3) a signal detector. Biosensors have become an important tool for the rapid, sensitive, and selective detection of microorganisms. These methods include biosensor-based electrochemical methods [16,17,18,19,20,21,22,23,24,25], fluorescence detection methods [20,21,22,23,24,25,26], and spectroscopy methods [27,28,29,30,31,32,33,34,35,36,37,38,39]. However, most of the biosensing methods require labeling of target objects for signal reading, which significantly increases the measurement time and cost. Moreover, the presence of dyes and labels tends to interfere with the normal physiological function of bacteria, which does not reflect the true state of the bacteria, especially in the evaluation of antibiotic resistance. Therefore, label-free methods are advantageous in rapid pathogen detection and drug resistance evaluation.

Compared with the labeling methods, which generally require a long incubation time, label-free approaches are much simpler, faster, and cost-effective, making them good candidates for rapid bacterial detection in clinical application. Efforts have been made in this direction, among which the optical methods, such as Raman spectroscopy and single-particle imaging approaches, are the most promising approaches due to their high sensitivity, simplicity, and low-cost for label-free detection of bacteria [40,41,42]. In this review, we describe the advantages and disadvantages of optical methods such as Raman spectroscopy, SPR, and dark-field microscopy for label-free detection of bacteria and their applications in clinical detection and drug resistance evaluation.

## 2. Surface Plasmon Resonance for Bacteria Detection

### 2.1. Principle of Surface Plasmon Resonance

**Figure 1:**
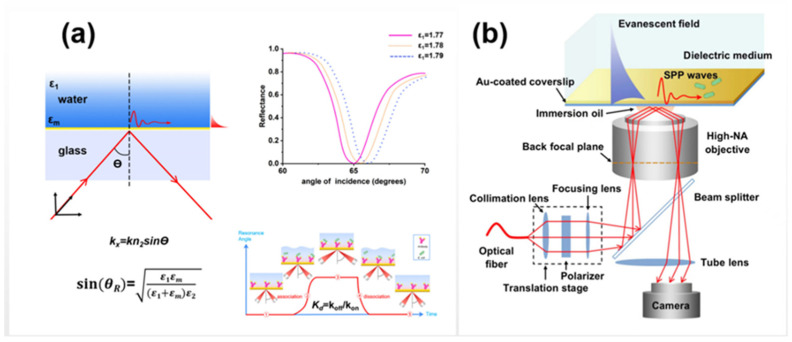
Schematic diagrams of (**a**) SPR optical system and (**b**) SPR microscopy.

A typical optical system of planar SPR is mainly composed of a polarized excitation light source, a prism and a glass sensor chip coated with a thin gold film (~50 nm). The incident light passes through the prism in total internal reflection mode. The reflected light significantly decreases at a specific angle (defined as the resonance angle), while the wave vector matches the surface plasma frequency of the gold film in the propagation direction as shown in Figure 1a. The shift of the SPR angle is very sensitive to the refractive index change at the metal–liquid interface, making it a powerful tool for real-time monitoring of molecular and particle binding at the interface in a label-free manner [43]. It has been used to analyze binding specificity between molecules [44,45,46], the concentration of target molecules [47,48], kinetic parameters of association and dissociation [49,50], etc. More recently, with the development of SPR microscopy as shown in Figure 1b, which can directly monitor the nanoscale motion of single bacteria at the interface, SPR microscopy has become a powerful tool for rapid drug resistance evaluation [51]. In contrast to conventional SPR biosensors such as BIAcore, which provide an average signal of the designed area on the surface of the sensor chip, SPR microscopy enables the detection of areas or particles of interest on the chip surface, facilitating the detection of bacteria at the single cell level This process can be accomplished by recording an SPR image of the chip surface with a charge-coupled device (CCD) or a complementary metal oxide semiconductor camera. In addition, high spatial resolution of the perceived surface can be obtained by introducing a lens or a high numerical aperture (NA) objective into the SPR image system to replace the prism [52,53]. In addition to SPR microscopy, the use of an SPR image to detect bacteria has also been widely reported. For example, Tripathi et al. [54] proposed coating the gold surface of traditional SPR biosensors with graphene to improve the adhesion of bacteria on the surface of the sensor and applied it to the detection of *Pseudomonas* and *Pseudomason*-like bacteria. Park et al. [55,56] immobilized antibodies onto the sensor chip via EDC mediated coupling and realized the label-free and highly sensitive detection of foodborne *Salmonella* at low PH (4.6) and high antibody concentrations (up to 1000 μg/mL).

### 2.2. Method and Application of SPR Technology for Label-Free Detection of Bacteria

The direct detection of bacteria by SPR requires specific antibodies against the target bacteria, which are immobilized on the surface of the gold film and specifically bind to the target bacteria to generate SPR signals. When the bacteria-containing solution flows to the sensor surface with specific antibody immobilization, the target bacteria bind to the gold film, which is then flushed to remove nonspecific interaction. As the SPR signal is positively correlated with the concentration of target bacteria, the number of target bacteria can be determined by setting up a calibration curve of bacterial concentration versus SPR signal intensity. The immobilization of antibodies on the sensor surface is a critical step for the detection of bacteria, which can improve the sensitivity and selectivity of bacterial SPR detection [57]. Physical adsorption and covalent binding are the main methods to fix the antibody on the sensor surface.

(i) Physical adsorption. Physical adsorption is a simple method of coating a surface that utilizes non-covalent bond interactions such as van der Waals forces, hydrogen bonds, electrostatic forces, and hydrophobic interactions to adsorb the target to the detection chip. Capturing bacteria on the surface creates a refractive index (RI) change, and RI is used to quantify the presence and quantity of the bacteria. Jarvis et al. [58] used SPR technology to track in real time the attachment of *Pseudom onas aeruginosa* bacteria to bare gold film. This study showed that the adsorption of wild-type and mutant bacteria and the concentration of bacteria in bacterial suspension could be distinguished by physical adsorption. The results of this method were compared with those of crystal violet assay for different mutant bacteria, and it was found that there was qualitative correlation between them. Another method of physical adsorption of bacteria is to first modify hydrophobic or hydrophilic compounds or biologically active molecules on the surface of the gold chip, and then incubate the bacteria with the modified surface of the gold sheet, so that it can be adsorbed to the surface of the gold chip in a non-covalent interaction. Livache et al. [59] used pyrrole co-electropolymerization to attach different types of carbohydrates to the surface of gold film. Because different carbohydrate types have different physical adsorption capacities compared to the five closely related *E. coli* strains, different types of *E. coli* were incubated and grown on the substrates modified with different carbohydrate strains. SPR imaging was used to detect their interactions with bacteria during culture. This method can detect and identify tested bacteria from an initial bacterial concentration of 10^2^ CFU/mL.

(II) Covalent immobilization. The measurement of SPR is based on the change of refractive index. However, because the gold film itself is not selective, it is not possible to distinguish the target in the complex mixture directly on the gold chip. SPR sensors specific to an analyte can be obtained by grafting an antibody that is specifically recognized by the analyte onto the surface of the gold chip. A reasonable method of immobilization of antibodies is to chemically conjugate antibodies to the surface of the sensor; immobilization of antibodies based on self-assembled monomolecular membrane (SAM) is the most studied method at present. SAM is an ordered single molecular structure formed by the adsorption of mercapto, amine, silane, or carboxylic acid components onto the solid surface in solution [43]. SAM can help control antibody binding direction, reduce nonspecific adsorption, and provide stable and directed analyte curing [60]. Thiolate compounds with different properties can easily be prepared with monolayers of different surface properties (such as wettability). SAM can be covalently bound to the primary amine of the ligand when it contains a carboxyl group at its end. This coupling is widely used for protein fixation. During the covalent binding of ligands, the non-specific binding of ligands on gold chips hinders the active functional groups in SAMs, which reduces the specificity. Therefore, a blocking agent, such as ethanolamine, is used to block the carboxyl groups remaining on the surface. In addition, bovine serum albumin is commonly used to block the gold surface to reduce the nonspecific interaction. Srikhirin et al. [61] developed an immunosensor based on SPR imaging using specific monoclonal antibody 11E5 (MAb 11E5) for the detection of seed-borne bacterium Acidovorax avenae subsp. citrulli (Aac). Aac was detected by self-assembly of MAb 11E5 mixed with monolayers (SAM). This method can be applied to multiplex detection, and it shows good selectivity for Aac with a limit of detection (LOD) of 10^6^ CFU/mL. Evoyet et al. [62] used cysteine labeling and mercaptan chemistry to modify a specific caudate protein (tsp) on the surface of gold film for specific capture of *Salmonella typhi* with a detection limit of 10^3^ CFU/mL. Chen et al. applied polyclonal anti-*E. coli* O157:H7 antibody to an NHS/EDC-activated surface by activating a SAM-coated chip with a mixture of NHS and EDC to generate an NHS ester receptor capable of binding to the amino group of the antibody via an amide bond [63]. Roupioz et al. used an antigen–antibody fixation method to modify the antibodies of a series of different bacteria in different regions of the gold sheet, and then cultured the advantages of this microarray on the chip with contaminated food. The culture of the bacteria results in an increase in the concentration of the target bacteria around the specific antibody, and then surface plasma resonance imaging is used to detect the growth of the bacteria. This single-step assay method enabled multiplex testing of *Cronobacterium* and *Salmonella* in less than a day and demonstrated that both bacteria were detected in 25 g of milk powder with as few as 30 CFU cells [64].

**Figure 2:**
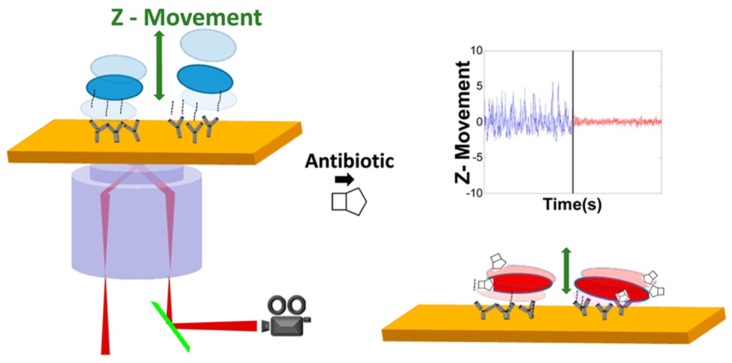
Schematic diagram of the rapid antimicrobial susceptibility test at single bacteria level using SPR microscopy [51]. Adapted with permission from Ref. [51]. Copyright © 2015 American Chemical Society.

Tao et al. modified the gold chip with a layer of PEG/PEG-COOH self-assembled monomolecular layer, and then activated PEG-COOH by NHS and EDC to generate NHS ester receptors that react with the primary amine group on the antibody by amide bonds. The polyclonal anti-*E. coli* O157: H7 IgG antibodies have been applied to NHS/EDC-activated surfaces so that bacteria can be specifically attached to the surface. By using SPR microscopy, the nanoscale-motion of bacteria can be sensitively monitored at the gold chip surface as show in Figure 2. As the nanoscale-motion of bacteria is related to their activities, Tao’s group developed a culture-free antimicrobial susceptibility test (AST) by tracking the motion using SPR microscopy, facilitating rapid antimicrobial resistance testing [51].

In addition to the above commonly used bacterial label-free detection, new methods have also been developed in recent years for SPR methods for bacterial label-free detection. Culture–Capture–Measure (CCM): The protein is covalently bound to the pyrrole monomer on the chip, and then different types of antibodies are modified on the chip in the form of microarrays. Bacteria are cultured on the surface of the chip and then combined with sensitive SPR assays, which enables rapid and specific detection of bacteria on the protein microarrays. This culture–capture–measurement method can significantly reduce the processing steps of bacterial detection and the overall analysis time of bacterial detection [64,65,66]. For example, Thierry et al. combined microbial incubation on chips with SPR detection to achieve rapid specific detection of *Salmonella enterica* serovar Enteritidis, *Streptococcus pneumoniae* and *E. coli* O157:H7 cultured on protein microarrays [65]. Several methods have also been proposed to further improve the sensitivity: A highly sensitive sensor based on surface material modification was constructed by modifying nanomaterials [67,68,69] (graphene, molybdenum disulfide, barium titanate) or organic compounds [59] (carbohydrate) on the surface of a gold chip, which can significantly improve the sensitivity of bacterial detection. Livache et al. detected their interactions with bacteria by efficiently grafting simple carbohydrates onto the surface of a gold sheet and then using surface plasma resonance imaging during the process of culturing the bacteria on the surface. It was found that each type of bacteria interacts with carbohydrate chips in different ways. Compared with the detection limit of 1.0 × 10^4^ CFU/mL for other electrochemical methods, the detection limit of this method can reach 1.2 × 10^2^ CFU/mL [59].

Besides the antibodies, the surface of the gold chip is modified with small molecules such as bacteriophages, polymyxin B, aptamers, etc., as a bacterial identification element [69,70,71]. For example, Michel Meunier et al. used l-cysteine SAM to coat a gold sheet, and then linked the T4 bacteriophage and BP14 bacteriophage to the self-assembled membrane respectively to specifically detect *E. coli* and Methicillin-resistant *S. aureus*. This method does not require the prior step of labeling or enriching bacteria and can detect concentrations of 10^3^ CFU/mL in less than 20 min [69]. In addition, the target bacteria were isolated and purified from complex samples by magnetic separation technology before SPR detection. Veli et al. developed a rapid and efficient magnetic separation step followed by the rapid detection of *B. melitensis* contamination in milk samples by SPR. Two aptamers with high affinity and specificity for *B. malitensis* were selected by a complete bacteria-SELEX procedure. The high-affinity aptamer (B70 aptamer) was immobilized on the surface of magnetic silica core-shell nanoparticles for the initial purification of target bacterial cells from the milk matrix. Another aptamer with high specificity for *B. melitensis* cells (B46 aptamer) was used to prepare SPR sensor chips for the sensitive determination of Brucella in magnetic purification eluted samples. This method can rapidly detect *B. melitensis* contamination in 1 mL milk samples by SPR, with LOD values as low as 27 ± 11 cells [72].

## 3. Raman Spectroscopy for Pathogen Bacteria Detection

### 3.1. The Principle of Raman Spectroscopy

Raman scattering can be defined as the inelastic scattering of photons from molecules. For every 10^6^ photons scattered from the molecules, approximately one photon is inelastically scattered (Raman scattering). The detection of inelastic scattering photons from a molecule produces a spectrum of Raman shifts by the acquisition of energy differences from incident light. Each Raman shift corresponds to a specific vibration mode of molecular bonds, thus allowing molecular identification based on a specific vibrational fingerprint. Compared to fluorescence spectroscopy, Raman spectroscopy has higher resolution and narrower bandwidth, making it easy for the multiplex detection of different analytes. An advantage of Raman spectroscopy for bacterial detection is that Raman scattering can occur at any wavelength. This allows free choice of the excitation wavelength to meet the needs of biological Raman spectroscopy acquisition, especially in reducing the significant background from fluorescence. Raman excitation using visible wavelengths can be integrated into standard light microscopes. This shorter wavelength excitation allows higher spatial resolution compared with infrared microscopy, allowing smaller sample volumes or even the detection of individual bacteria.

### 3.2. Label-Free Detection of Bacteria by Raman Spectroscopy

Raman spectroscopy has been used for many years to probe the biochemistry of various biomolecules, and more recently for disease detection. Specifically, Raman spectroscopy has been used to characterize bacteria in microbial colonies to detect their presence in smaller sample sizes with rapidity. However, most bacterial detection using RS relies on microspectral identification of reference strains or clinical isolates [73,74,75]. Raman mic rospectroscopy can detect bacterial cells in liquid suspensions, and it can identify bacteria directly from patient body fluids without culture. Sandra et al. conducted two studies in which isolation protocols from filtration [76] and centrifugation [77] were both developed to extract bacteria from patient sputum and urine, respectively. The type of causative strain was determined by Raman spectroscopy. By combining Raman spectroscopy with hierarchical cluster analysis (HCA), Jiirgen et al. directly detected individual bacterial cells from cerebrospinal fluid samples of meningococcal patients without any sample preparation steps [78].

The major limitation is that Raman scattering is extremely weak, resulting in relatively poor sensitivity compared with other optical methods such as autofluorescence and absorption [78]. This means that collecting vibrational spectra via spontaneously generated Raman photons requires extremely sensitive detection hardware, long exposure times, and relatively high excitation power compared to other optical techniques. In recent years, surface-enhanced Raman spectroscopy (SERS) has been extensively studied in the detection of chemical and biological agents with its rapid and ultra-sensitive characteristics [79,80].

### 3.3. Label-Free Detection of Bacteria by SERS

SERS is a combination of Raman spectroscopy and nanotechnology. It retains the advantages of fast acquisition of RS, less sample consumption, and fingerprint spectra for specific analytes. In addition, SERS significantly enhances the sensitivity of Raman spectroscopy over several orders, thus reducing the interference from self-fluorescence. The weak Raman scattering intensity of the sample is greatly enhanced by placing the sample on the nanoscale rough noble metal surface or mixing the sample with the noble metal colloidal suspension. In SERS, the average enhancement coefficient was between 10^4^ and 10^8^, and it could reach 10^11^ in some cases [81,82,83,84].

The SERS effect can be explained by two enhancement mechanisms: electromagnetic and chemical. The former is the enhancement of electromagnetic field due to local surface plasmon resonance (LSPR) [85,86], while the latter is chemical enhancement due to the charge transfer process between metal nanoparticles and analytes [86], although the contribution of this mechanism has been shown to be much lower than that of electromagnetic enhancement. Two SERS methods have been developed, the label-based method and the label-free method. However, despite the high sensitivity, label-based methods only provide information about reporter molecules and lose the intrinsic information of bacterial cells. The accuracy of the label-based method is entirely dependent on the specificity of recognition molecules. In addition, the labeling will significantly increase the sample analysis time. Compared with the label-based SERS method, the label-free method is rapid and easy to operate without any external labeling [87]. Label-free methods can detect bacteria by measuring the SERS pattern inherent in the cell wall, allowing for direct bacteria identification. However, the sensitivity of the label-free SERS method largely depends on the SERS substrate, the bacterial species, and the sample preparation methods.

Noble metal nanoparticles such as gold and silver are the usually preferred light intensifiers in SERS. The plasmonic characteristics of these noble metal nanoparticles, namely LSPR and the electromagnetic field generated on the surface, are mainly determined by the size, shape, and mutual assembly of the metal nanoparticles and the dielectric properties of the surrounding medium [88]. In general, silver-based SERS substrates have higher SERS enhancement effects than gold. However, silver is less stable, and has a biotoxic effect on living organisms, which limits its application in living organisms. Gold is much more stable, strongly chemical inert, and less biotoxic than silver. The nanostructure of gold is stable, facilitating better control of the size and shape of particles with higher biocompatibility. In order to achieve highly sensitive and repeatable SERS detection, the size, shape, and stability of metal particles should be reasonably controlled. The aggregate of nanoparticles was found to exhibit a larger Raman-enhanced signal than individual nanoparticles due to the generation of hot spots in the gaps between nanoparticles. Additionally, the nanostructure with sharp tips can also significantly enhance the SERS intensity. The generation of hot spots is highly sensitive to the size, shape, and gap-distance of nanoparticles [87]. Therefore, top-down lithography methods and bottom-up self-assembly methods have been developed to control the shapes, arrangements, and assemblies of nanoparticles [89,90,91].

In general, there are several strategies that have been developed for the direct label-free SERS detection of bacteria, which are summarized as follows.

#### 3.3.1. In Situ Formation of Colloidal Silver/Gold on the Surface of Bacteria

The common methods for forming colloidal silver/gold on the surface or inside of bacteria are achieved by soaking the bacteria in sodium borohydride solution, then resuspending in silver nitrate or chloroauric acid (HAuCl_4_). The metal ions outside the cell wall react with reducing agents released from the cell, resulting in the colloids formation on the cell wall. Tamitake et al. employed a focused near-infrared laser beam to capture individual bacteria in aqueous Ag nitrate; Ag nanoaggregates were generated on *E. coli* by an additional green laser beam stimulation. In this way, the Raman scattering signal of *E. coli* was obtained by the Raman tweezer technique at single cell level [92].

#### 3.3.2. Direct Bacteria Detection on a Planar SERS Surface

The planar SERS substrate can be gold-plated glass slides with high roughness or self-assembled SERS active substrate through rational design. The bacterial suspension is dropped on the substrate and allowed to dry for bacterial detection [93,94,95]. Wang et al. [96] prepared Ag/AAO SERS substrates embedding Ag nanoparticles in anodic aluminum oxide (AAO) nanochannels. This substrate possesses high reproducibility, therefore can be further analyzed by principal component analysis (PCA), linear discriminant analysis (LDA), and support vector machine (SVM) to detect Staphylococcus Aureus (Gram-positive bacterium), Klebsiella Pneumoniae (Gram-negative bacterium), and Mycobacterium Smegmatis (Mycobacterium) and other bacteria, providing a good strategy for clinical microbial detection.

**Figure 3:**
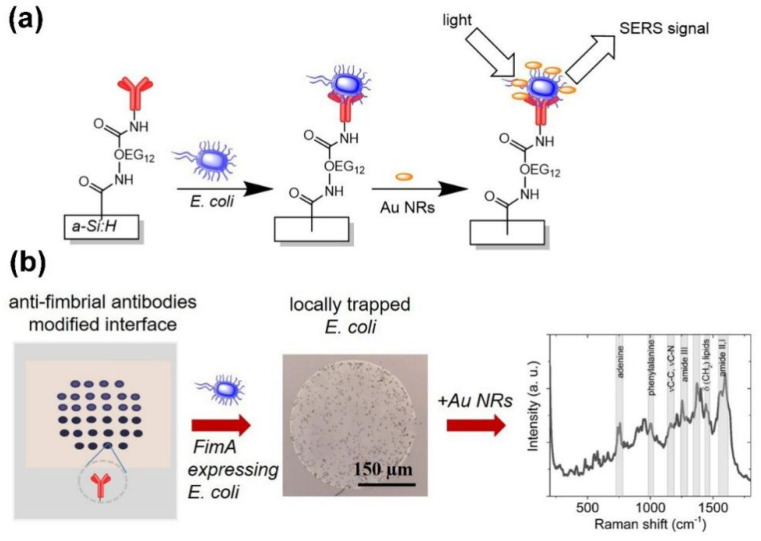
Schematic detection principle of *E. coli* hydrogenated amorphous silicon a-Si:H surface modified with anti-fimbrial antibodies against the major pilin protein fimA. (**a**) Surface structures of *E. coli* expressing fimA selectively captured and positively charged Au-NRs incubated with *E. coli* for SERS sensing. (**b**) Anti-fimbriae modified array, optical imaging of spots after interaction with *E. coli* and SERS spectra after capturing bacteria [97]. Adapted with permission from Ref. [97]. Copyright © 2020 Elsevier B.V.

Andrei et al. reported that with the modification of anti-fimbrial antibodies onto the polyethylene glycol (OEG12) molecular layer on the amorphous hydrogenated silicon (a-Si:H) film. The fimbriated *E. coli* was specifically captured onto the surface as shown in Figure 3a. The positively charged gold nanorods (Au NRs) were attracted to the negatively charged *E. coli* on the film, facilitating the reading of the SERS signals. This method has high repeatability for the detection of bacteria, due to the uniform coverage of Au NRs on the bacterial membrane [97].

**Figure 4:**
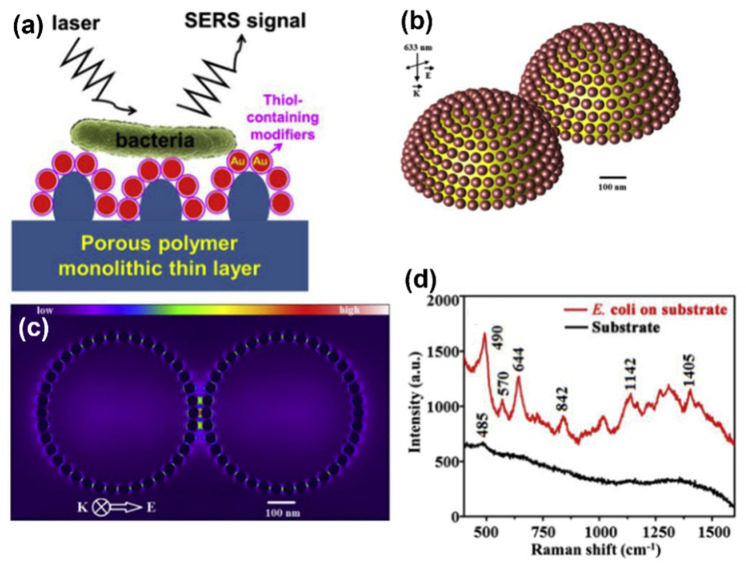
Schematic and detection principle of GNP/monolith modified substrate for the capture of *E. coli*. (**a**) Cross-sectional view of *E. coli* captured on gold nanoparticles modified substrates. (**b**) SERS enhancement factor of porous substrate functionalized with 40 nm gold nanoparticles simulated by FDTD. (**c**) In the simulation, the geometry of the model is reduced to two hemispheres coated with 40 nm spherical gold nanoparticles, separated by 10 nm; the electric field intensity distributions in x-y plane and y-z plane of gold on porous monolithic substrate excited by 633 nm laser are calculated. (**d**) SERS spectra of 40 nm gold nanoparticles/substrate functionalized with cysteamine [98]. Adapted with permission from Ref. [98]. Copyright © 2015 Elsevier B.V.

Lv et al. used glycidyl methacrylate and ethylene dimethacrylate to prepare a convex substrate using a concave glass mold. The surface was treated with mercaptan to capture the Au nanoparticles on the surface as shown in Figure 4. The bacterial suspension is dropped on the SERS substrate, and the SERS spectrum of *E. coli* can be obtained after the sample dries naturally, as shown in Figure 4d. This simple SERS substrate preparation method proposed in this study was able to generate homogeneous and reproducible SERS active substrates over a large area, which has significantly improved the sensitivity of Raman spectroscopy. In this experiment, propanethiol, 3-mercaptopropionic acid, and cysteamine were modified on the surface of gold nanoparticles to improve the preferential adsorption ability of bacteria in very diluted thallus solution, while the SERS spectrum was used for the direct detection of the captured microorganisms as shown in Figure 4d [98].

#### 3.3.3. Direct Bacteria Detection in SERS Suspension

Bacteria detection can be achieved in the suspension by directly mixing the bacteria with colloid. By optimizing the volume ratio of bacterial suspension to colloidal silver. Davis et al. were able to detect *E. coli* as low as 10^3^ CFU/mL by correcting the Raman spectrum of the wide vibrational OH band in water [99]. Jennifer developed a bacterial SERS detection platform that can detect bacteria in a controlled liquid environment that maintains the viability of bacteria in a liquid environment. Plasmon resonance nanorods with different longitudinal lengths were used to detect Gram-negative *E. coli*, *Staphylococcus epidermidis*, *Serratia marcescens,* and Gram-positive *S. aureus*. The SERS signal was much higher with the higher surface charge density of the bacteria, indicating that the higher SERS-enhanced signal comes from the electrostatic attraction between the positively charged nanorods and the negatively charged bacteria. This label-free liquid-SERS assay provides a promising strategy for bacterial identification and AST testing in living organisms [100].

## 4. Label-Free Detection of Bacteria by Dark-Field Microscopy

### 4.1. Dark-Field Microscopy Imaging Principle

Dark-field microscopy is a microscopy technique that obliquely illuminates a sample by attaching a circular opaque baffle to a condenser to prevent the incident light from directing into the camera [101]. When the incident light enters the condenser, the center part is blocked by the baffle, leaving the edge light to pass through. The annular beam formed by the incident light turns into a hollow conical beam after the light is concentrated through the condenser, and illuminates the sample, thus stimulating the scattering of sample particles. In this setting, only scattering light from objects in the medium enters the objective lens, creating a bright scattering pattern in a dark background [102]. Due to the Tyndall effect, particles far below the resolution limit of typical light microscopes can be observed using dark-field microscopes [103].

### 4.2. Label and Label-Free Detection of Bacteria by Dark-Field Microscopy

**Figure 5:**
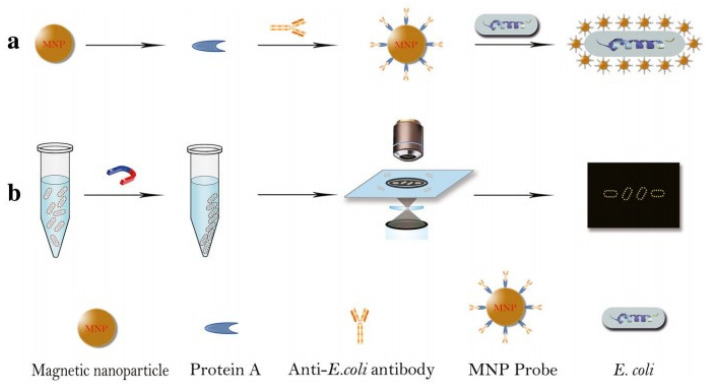
Schematic diagram of counting *E. coli* under dark-field, using antibody functionalization of MNP to form a gold ring structure around *E. coli.* (**a**) MNP probe was obtained by culture of *E. coli* antibody onto MNP. *E. coli* samples are first mixed with MNP probes to form probe-*E. coli* complexes. (**b**)The complex of *E. coli* and MNP probes was separated by a magnet and then counted under a dark-field microscope. [119]. Adapted with permission from Ref. [119]. Copyright © 2018 The Author(s).

Dark-field microscopy is an interesting optical technique that has been successfully used to image bacteria [40,104,105,106,107,108,109,110,111,112,113,114] and protozoa [102,115] due to its very low background, simple construction, portability, and low cost. Since plasma nanoparticles exhibit strong scattering to visible light, dark-field microscopy is a powerful tool for imaging and localization of noble metal nanoparticles in single cell analysis [101,109,116,117]. For example, hollow gold-silver nanoparticles are used as an alternative, less invasive contrast agent to assess the uptake process of malignant lymphocytes [118]. When the nanoparticles were modified by ligand and specifically bound to the cell membrane or internalized into the organelles, bright spots of different sizes and strengths could be observed on the surface of the target bacteria or around the organelles. Bacteria can be identified or counted based on the location and intensity of the bright spots. For example, Li et al. [104] developed a simple and fast bacterial count method based on dark-field light scattering imaging of a bacteria using gold nanoparticles as reporters. Zhou et al. [119] functionalized magnetic nanoparticles (MNP) using specific antibodies, which then formed a ring structure around *E. coli*, facilitating the counting of MNP conjugated *E. coli* under a dark-field microscope, as shown in Figure 5. In a similar way, Watanabe et al. [112] used phages as biometric elements, and aggregation-induced light scattering signals from silica nanospheres assembled by gold nanoparticles as signal transducers. After mixing the samples with the phage scattering probe of *S. aureus*, the detection limit of *S. aureus* was 8 × 10^4^ CFU/mL within 15–20 min.

Shiigi et al. [117] developed a novel molecular imprinting polymer (MIP) particle coated with gold nanoparticles (AuNPs) that can act as an acceptor and an optical signal transmitter in biological systems after modifying specific antibodies on its surface. Due to the coating of AuNPs, MIP particles produce a strong scattered light signal, and the binding of MIP particles increases the light intensity of the target bacteria. This allows bacteria to be clearly visible under darkfield microscopy, allowing them to be quantified using scattered light intensity. Using this technique, they successfully quantified *E. coli* O157 cells in meat samples.

**Figure 6:**
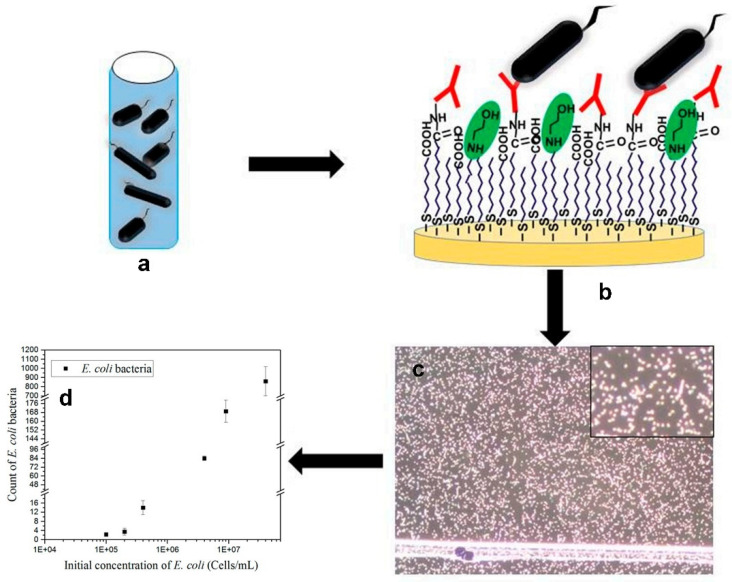
Schematic of detection of *E. coli* with dark-field microscopy. (**a**) Samples containing *E. coli.* (b) an anti-*E. coli* antibody functionalized gold surface. (**c**) Dark-field microscopy is used to inspect the surface of the gold sheet after 75 min incubation with the field sample and rinse with phosphate buffer solution, enlarging the image. (**d**) Statistical image analysis was used to count the bacteria captured by the antibodies [40]. Adapted with permission from Ref. [40]. Copyright © 2019 MDPI.

Although powerful, the above-mentioned methods require the use of nanoparticles for signal reading of bacteria via dark-field microscopy, which affects the original physiological activity state of the bacteria detected and cannot reflect the real physiological activity and quantity of the bacteria [40,117,120,121,122]. Therefore, it is more desirable to detect bacteria in a label-free, rapid manner as the scattering intensity of bacteria is strong enough for direct dark-field imaging. In recent years, several methods have been developed to detect bacteria label-free using dark-field microscopy. For example, Colpo et al. [40] established a sensing platform for the rapid detection of bacteria in field samples using specific antibodies as recognition elements and dark-field microscopy as detection technology. By covering a gold layer on the polished silicon wafer and covalently modifying polyclonal anti-*E. Coli* antibodies to the surface, the sensing chip can be used for the specific capture of *E. coli* on the surface. As shown in Figure 6, the circularity and size of the object were used to identify the captured bacteria by dark-field microscopy. The performance was tested and compared to the Colilert-18 test and the quantitative polymerase chain reaction (qPCR), which showed comparable results.

Creighton et al. identified Treponema Pallidum under optical microscopy with double-reflection and single-reflection dark-field condensers based on spirochetes of bacterial characteristic morphology and locomotion criteria. Ideally, this method can identify *Treponema Pallidum* using dark-field microscopy within 20 min [120].

**Figure 7:**
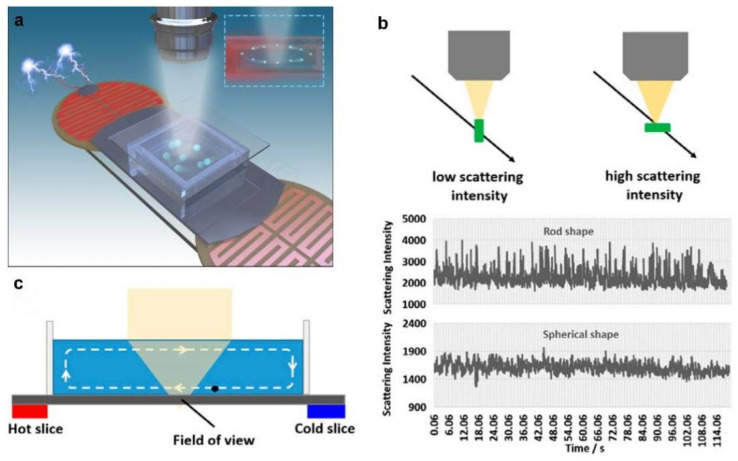
Bacteria detection principle by a single-particle imaging approach. (**a**) Schematic diagram of bacteria detection by single-particle imaging. (**b**) The inhomogeneity of particle morphology is identified by tracking the fluctuations of scattering intensity in free solution. (**c**) Convection induced by an electric heater was used to screen individual bacteria in a small field of view [121]. Adapted with permission from Ref. [121]. Copyright © 2022 The Author(s).

Rapid diagnosis of bacterial infectious diseases has important clinical significance for rapid and rational use of antibiotics, so as to avoid the misuse of antibiotics. However, the detection of pathogenic bacteria generally requires molecular identification using antibodies or aptamers, which requires long incubation time, as well as complex sample pretreatment and signal amplification. To address this challenge, Fang [121] and Wang [122] used light scattering imaging methods to detect individual bacteria without labeling by the scattering intensity trajectory of particles in free solution. The scattering strength variation provides particle shape information because it is relevance to the morphological heterogeneity of the particle. The fluctuating pattern of the scattering intensity also depends on the shape and orientation of the particles in free solution, such as rod-shaped bacteria, whose scattering intensity fluctuates significantly higher than that of the spherical shape in free solution, which can be used to characterize the shape of the bacteria. Fang’s group used label-free single-particle dark-field imaging for rapid and sensitive identification of bacteria in free solution by modulating the convection [121] as shown in Figure 7. Using this method, they were able to distinguish positive samples of streptococcus agalactiae from vaginal swabs within 10 min without the use of any biological reagents. In addition to the spherical shape bacteria, the optical characteristics of single bacteria with different shapes such as *E-coli* are also significantly different from the matrix, implying that the rapid detection of different types of bacteria in one clinical sample is plausible, facilitating the precise prescription of antibiotics.

**Figure 8:**
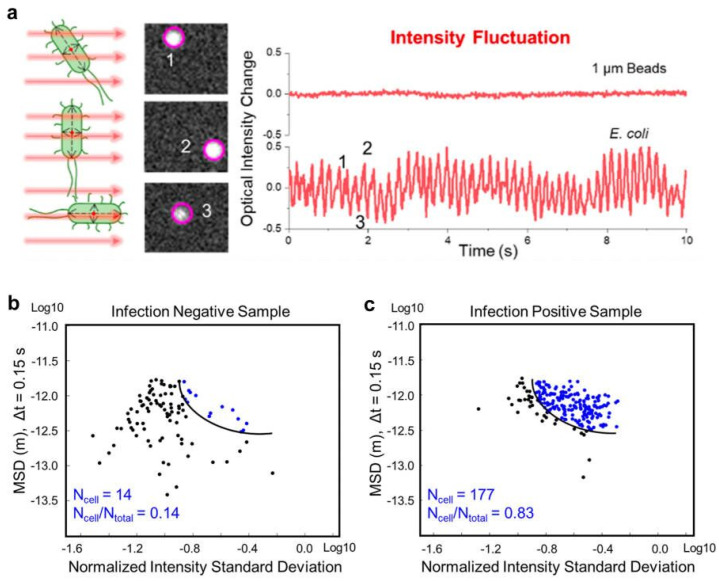
The principle of tracking the rapid identification of 1 um polystyrene spheres and single cell phenotypic characteristics of *E. coli*. (**a**) *E. coli* rotation-induced scattering intensity fluctuation tracking compared with 1 µm microbeads. (**b**) SVM classification result of one representative infection negative sample. (**c**) SVM classification result of one representative infection positive sample. [122]. Adapted with permission from Ref. [122]. Copyright © 2022 American Chemical Society.

Similarly, Wang et al. used a large-volume solution scattering imaging (LVSi) system to track the scattering intensity and movement track of individual bacteria in short videos. The machine learning algorithm was used to perform aggregation analysis on their scattering intensity and movement trajectory. The presence of *E. coli* or similar bacteria in urine could be accurately determined, and bacteria could be distinguished from other common particles in urine, as shown in Figure 8. The method can detect patients with urinary tract infection within 10 min with an accuracy of 92.3%.

## 5. Other Methods for Label-Free Detection of Bacteria

**Figure 9:**
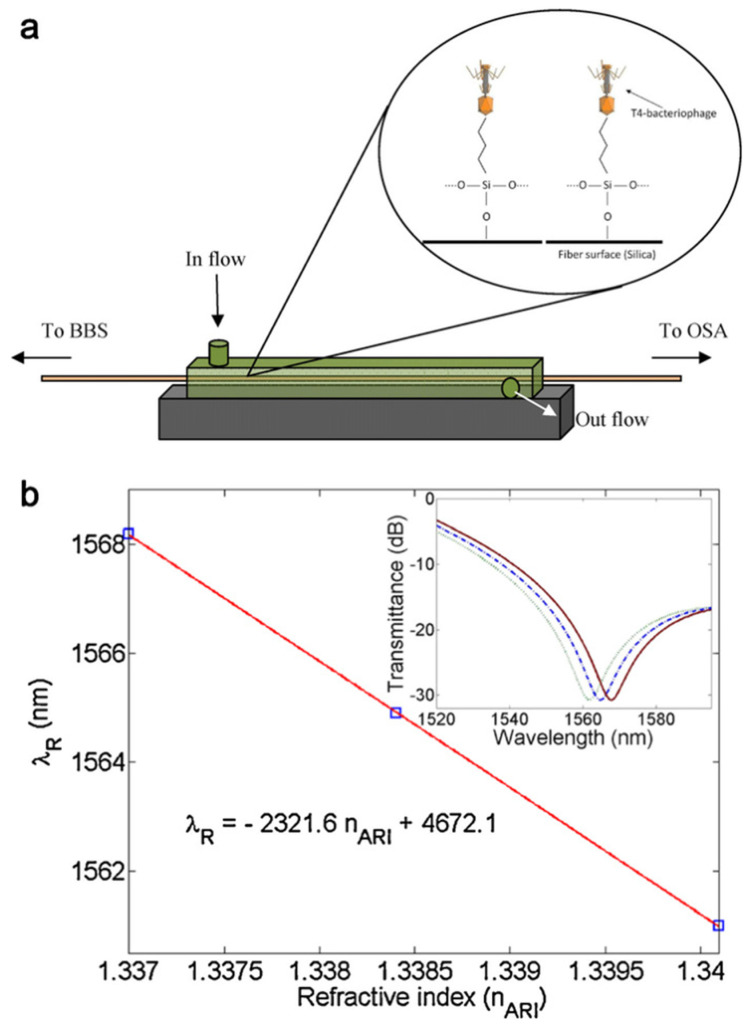
Schematic illustration of the experimental arrangement. (**a**) Covalent binding of phage to SiO_2_ on fiber surface. (**b**) Resonance wavelength change with analyte refractive index transmission spectrum [124]. Adapted with permission from Ref. [124]. Copyright © 2012 Elsevier B.V.

Other progress in the field of label-free optical biosensors is the advent of optical fiber gratings. Smietana [123] et al. first proposed a low-cost LGPs sensor that detects specific *E. coli* without labeling by physical adsorption. To further improve the sensitivity, Saurabh [124] proposed a compact ultra-sensitive long-period fiber grating (LPFGs) detection method for high-sensitivity label-free detection of specific *E. coli*. with modification of bacteriophage as shown in Figure 9. Simona [125] developed a reflective long-period fiber grating (RT-LPG) biosensor that can rapidly detect Class C β-lactamases in simple and complex biological samples. Additionally, fiber Bragg gratings (FBGs) can be used for bacterial detection [126,127].

Alternatively, the bacteria can be detected with the preparation of SERS hot spots on a fiber tip using optical fiber technology. The fiber-optic SERS probe (SERS on-a-tip) is highly controllable and reproducible [128,129,130].

Similarly, Biolayer interferometry (BLI) technology has been reported for bacterial detection in recent years. BLI is a label-free optical detection technique for real-time monitoring of biomolecular interactions. When an analyte binds to a ligand immobilized on the tip surface of a glass fiber-optic biosensor, its spectrum shifts with the change in the thickness of the related molecular layer. For example, Zhang et al. [131] reported a new method for the rapid, label-free real-time detection of *Salmonella enterica* using NLI incorporating antibodies as receptors, with a detection limit of 1.6×10^5^ CFU/mL. Gu et al. [127] used C54A mutant LysGH15 as a receptor and combined it with BLI to establish a rapid, highly specific and label-free method for real-time detection of *Staphylococcus aureus* (*S. aureus*). This method can directly detect *S. aureus*, and its detection limit is 13 CFU/mL.

## 6. Conclusions

In this paper, the applications of SPR, Raman spectroscopy and dark-field microscopy for the label-free detection of pathogenic bacteria are reviewed. The principle of SPR, Raman spectroscopy, dark-field microscopy as well as fiber-based methods for the label-free detection of pathogenic bacteria are considered. These label-free optical methods possess advantages of rapidity and low-cost, and are promising candidates for the clinical use for infectious disease diagnosis, facilitating the precise prescription of antibiotics to avoid the misuse of antibiotics, which is becoming a global problem.

The SPR imaging platform has been applied for high-throughput analysis, including the simultaneous detection of different bacterial species, antibiotic and bacterial interactions, etc. However, SPR generally suffers from the problem of non-specific adsorption and the direct detection of bacteria without sample preprocessing remains a challenge. Due to the high spatial resolution, SPR microscopy is able to image the bacteria at single cell level and possibly distinguish particles by their mass, and is potentially able to differentiate nonspecific adsorption. However, SPR microscopy is not commercially available, the total internal reflection fluorescence objective used for SPR microscopy is quite expensive. SERS is another label-free method for the rapid detection of bacteria with low cost based on the fingerprint vibration spectra. However, it is still a challenging task to detect bacteria in a label-free manner in complex biological environments. An obstacle lies in the large SERS background contributed from the complex matrix. Fortunately, recently developed machine learning methods are possibly to address this challenge. Direct detection of bacteria by dark-field microscopy on a substrate can be significant interfered with by nonspecific adsorption of other substances such as cell fragments and exosomes in the matrix, therefore, relatively few studies on the label-free detection of bacteria by this technique have been reported. However, the direct imaging of bacteria in free solution by dark-field microscopy is a unique approach reported recently which is quite promising in addressing this challenge due to it rapidity and low-cost, as it does not need any biological reagents or an incubation process. Despite the difficulties in differentiating bacteria with similar sizes and shapes, the recently developed image recognition and machine learning technologies are likely to address this challenge. Therefore, we believe that this dark-field imaging method for label-free bacterial detection in free solution will be widely used in bacterial detection, clinical diagnosis, and infectious disease control due to its high sensitivity, rapidity, simplicity, and low-cost.

Compared with the label-free optical methods, the paper based colorimetric methods have attracted increasing attention due to their simplicity and cost-effectiveness, as well as the rapid signal readout with the naked eye, making them a promising candidate for the development of point of care devices [132]. However, the colorimetric methods are largely compromised by relatively poor sensitivity. Signal amplification methods can be applied to further improve the sensitivity, but they require additional processes, which significantly increase the detection time. Therefore, we believe that the reagent-free dark-field imaging method for label-free bacterial detection in free solution is more advantageous and will be widely used in bacterial detection, clinical diagnosis, and infectious disease control due to its high sensitivity, rapidity, simplicity, and low-cost.
